# Assessing resources for implementing a community directed intervention (CDI) strategy in delivering multiple health interventions in urban poor communities in Southwestern Nigeria: a qualitative study

**DOI:** 10.1186/2049-9957-2-25

**Published:** 2013-10-24

**Authors:** Ikeoluwapo O Ajayi, Ayodele S Jegede, Catherine O Falade, Johannes Sommerfeld

**Affiliations:** 1 Epidemiology and Biostatistics Research Unit, Institute of Medical Research and Training, College of Medicine, University of Ibadan, Ibadan, Nigeria; 2 Department of Epidemiology and Medical Statistics, College of Medicine, University of Ibadan, Ibadan, Nigeria; 3 Department of Sociology, The Faculty of Social Sciences, University of Ibadan, Ibadan, Nigeria; 4 Department of Pharmacology and Therapeutics, College of Medicine, University of Medicine, University of Ibadan, Ibadan, Nigeria; 5 Special Programme for Research and Training in Tropical Diseases (TDR), World Health Organization, Geneva, Switzerland

**Keywords:** Community directed intervention, Urban poor, Health interventions, Community participation

## Abstract

**Background:**

Many simple, affordable and effective disease control measures have had limited impact due to poor access especially by the poorer populations (urban and rural) and inadequate community participation. A proven strategy to address the problem of access to health interventions is the Community Directed Interventions (CDI) approach, which has been used successfully in rural areas. This study was carried out to assess resources for the use of a CDI strategy in delivering health interventions in poorly-served urban communities in Ibadan, Nigeria.

**Methods:**

A formative study was carried out in eight urban poor communities in the Ibadan metropolis in the Oyo State. Qualitative methods comprising 12 focus group discussions (FGDs) with community members and 73 key informant interviews (KIIs) with community leaders, programme managers, community-based organisations (CBOs), non-government organisations (NGOs) and other stakeholders at federal, state and local government levels were used to collect data to determine prevalent diseases and healthcare delivery services, as well as to explore the potential resources for a CDI strategy. All interviews were audio recorded. Content analysis was used to analyse the data.

**Results:**

Malaria, upper respiratory tract infection, diarrhoea and measles were found to be prevalent in children, while hypertension and diabetes topped the list of diseases among adults. Healthcare was financed mainly by out-of-pocket expenses. Cost and location were identified as hindrances to utilisation of health facilities; informal cooperatives (*esusu*) were available to support those who could not pay for care. Immunisation, nutrition, reproductive health, tuberculosis (TB) and leprosy, environmental health, malaria and HIV/AIDs control programmes were the ongoing interventions. Delivery strategies included house-to-house, home-based treatment, health education and campaigns. Community participation in the planning, implementation and monitoring of development projects was reported as common practice. The resources available for these activities and which constitute potential resources for the CDI process include community volunteers, CBOs and NGOs. Others are landlords; professional, women and youth associations; social clubs, religious organisations and the available health facilities.

**Conclusion:**

This study’s findings support the feasibility of using the CDI process in delivering health interventions in urban poor communities and show that potential resources for the strategy abound in the communities.

## Multilingual abstract

Please see Additional file 1 for translations of the abstract into the six official working languages of the United Nations.

## Background

Neglected populations living in poverty throughout the developing world, such as the urban and the rural poor, are often heavily burdened by communicable and non-communicable diseases, and are highly marginalised by the health sector due to their limited access to health and social support services [1]. The population density and diversity of urban communities offers formidable challenges for healthcare delivery. The constant mobility (within urban areas, rural–urban–rural cycles) further complicates the delivery of appropriate health interventions. The current approaches and systems in urban areas have thus far been unable to reach agreed-upon goals and targets (e.g., the MDGs, RBM, national targets) and bridge existing gaps in health care [2]. Without improved delivery of health services, the present obstacles – accessibility, affordability and utilisation of the health systems – will perpetuate disparities and likely increase the risk factors, incidence and prevalence of treatable and manageable health conditions as the size of vulnerable and marginalised urban populations grows. Reduction in disease burden would enable these communities and groups to become more economically active and, thereby, further reduce the socio-economic factors contributing to disease occurrence.

Achieving reduction in disease burden lies in ensuring that available health interventions reach those who are at risk. Many simple, affordable and effective disease control measures have had limited impact due to poor access especially by the poorer populations (urban and rural) and inadequate community participation [3]. A proven strategy to address the problem of access to health interventions is Community Directed Treatment with ivermectin (CDTi) used for mass distribution of ivermectin in rural African populations for the control of onchocerciasis. This is based on the principle of active structural community participation [4]. The process empowers community members to make major decisions, and organise and direct the distribution of ivermectin for a sustained period of years. In order to improve access of Africa’s rural communities to other critical health interventions, the CDTi process was further tested for feasibility of delivering multiple interventions latching on ivermectin distribution. This project tagged 'Community Directed Interventions (CDI) for major health problems in Africa’ was found to be effective and efficient thus providing overwhelming evidence for its use as a strategy in delivering multiple interventions at the community level in rural Africa (CDI study group) [5].

While the urban areas have their own social structure and socio-cultural organisations, most urban local government areas (LGAs) in Nigeria are not likely to have the CDI strategy for ivermectin distribution in place because of the low incidence of and risk factors of onchocerciasis in urban communities. However, the CDI process offers a testable strategy in urban areas especially the urban poor where access to health care and interventions are inadequate. There is thus a need to test the feasibility, acceptability and effectiveness of the CDI strategy in delivering health interventions in under-served urban communities. However, implementing a CDI strategy in urban areas is faced with the challenges of determining what should be the appropriate approach to adopt, disease(s) to focus on and which resources are available in the community to support such a strategy. To this end, during 2011–12, the World Health Organization’s Special Programme for Research and Training in Tropical Diseases (TDR) sponsored a multi-country situation analysis in four large and medium-sized urban settings throughout Africa – including Ghana (Bolgatanga, Wa), Liberia (Monrovia), Nigeria (Ibadan) and the Democratic Republic of Congo (Kinshasa) – to explore the feasibility of the CDI approach in addressing multiple disease intervention in urban communities. This present paper is on the situation analysis carried out in Ibadan, Nigeria. The goal was to document the delivery process of existing health interventions and resources available in poorly-served urban communities in order to generate background information for planning to improve healthcare delivery in poorly-served urban communities through the CDI approach. To achieve this goal, the study set out to identify poorly-served urban communities, determine prevalent diseases and healthcare delivery services in those communities and, consequently, explore the potential resources available for the CDI approach.

## Methods

### Study design

The study employed a descriptive design, mainly based on a cross-sectional survey and qualitative methods, both standardised throughout the sites detailed in a multi-country core protocol.

### Study area

For the purpose of the study, an urban area was defined as a geographic area sharing the characteristics of a city, in which dwells a group of people who share common characteristics and interests. Only urban areas that have never had CDTi intervention were eligible for inclusion in the study.

The study was carried out in Ibadan city, the capital of Oyo State, which is made up of 33 local government areas (LGAs) (also referred to as districts in other African countries), each divided into a minimum of ten wards, the lowest political structure. Each ward consists of a population range of 10,000 to 20,000 people. The proportion of the rural/urban population of Oyo State is 31% and 69%, respectively [6]. Eleven of the 33 LGAs constitute Ibadan metropolis, which is further divided into core Ibadan [five LGAs – Ibadan North, Ibadan Southwest (SW), Ibadan Northwest (NW), Ibadan Southeast (SE) and Ibadan Northeast (NE)], and outer Ibadan [six LGAs – Lagelu, Ona Ara, Egbeda, Akinyele, Ido and Oluyole]. The five LGAs in core Ibadan are the only LGAs that are mainly urban in Oyo State while the six LGAs in outer Ibadan are both urban and rural (in different proportions).

The study LGAs were selected from the 11 LGAs that make up the larger Ibadan agglomeration using two-stage sampling techniques. Firstly, Ibadan was stratified into two: core metropolis and outer Ibadan. Secondly, two (2) LGAs were selected from each of the stratum by balloting. Four LGAs namely: Ibadan North, Ona Ara, Ibadan SW and Akinyele were selected. All the LGAs are predominated by the Yoruba ethnic group with other ethnic groups distributed in unequal proportions depending on the commercial activities in the LGA and its geographical location. The major religions are Christianity and Islam, but traditional religions also exist. Having appraised all the communities in the selected LGAs, eight urban, poorly under-served communities namely Moniya and Ojoo in Akinyele, Alagbafo and Inalende in Ibadan North, Foko and Odo Ona/Apata in Ibadan SW, and Olorunsogo and Olunloyo in Ona Ara were selected for the study. The profile of each of these communities is presented in Table 1.

**Table 1:** Profile of the selected study communities

**S/N**	**Communities**	**Number of health facilities**				**Population size (2010 projection)**	**Major source of water supply**	**Means of waste disposal**	**Means of faecal disposal**
		**Public**	**Private**	**Mission**	**Other**				
1	Moniya	6	4	0	0	28,990	• Well water is the major source	• Indiscriminate dumping at dump sites and drainages	• Most use latrine at home
2	Ojoo	2	13	0	0	35,737	• Well water is the major source	• Indiscriminate dumping at dump sites and drainages in some areas	• Most use latrine at home
								• Private refuse collector are engaged by some areas	
3	Alagbafo	1	1	0	1	22,635	• Well water and spring are the major sources of water although water pipe runs through but disconnected	• Refuse containers provided by the government	• Most use latrine at home
4	Foko	1	3	0	0	29,279	• Well water is the major source of water although water pipe runs through the community but irregular	• Indiscriminate dumping at dump sites and drainages in some areas	• Indiscriminate disposal of fecal matter through the drainage or dumping site
5	Inalende	0	3	0	1	24,279	• Well water is the major source of water although water pipe runs through the community but irregular	• Indiscriminate dumping at dump sites and drainages in some areas	• Most dispose their fecal matter through the drainage or dump site
6	Odo-Ona/Apata	4	7	2	0	55,429	• Pipe borne water irregular	• Indiscriminate dumping at dump sites and drainages in some areas	• Most use latrine at home
							• Well water is the major source		
7	Olorunsogo	1	6	0	2	38,475	• Well water is the major source	• Indiscriminate dumping at dump sites and drainages in some areas	• Most use latrine at home
8	Olunloyo	1	2	0	0	25,531	• Well water is the major source and some have borehole	• Indiscriminate dumping at dump sites and drainages in some areas	• Most use latrine at home

### Healthcare delivery system

All the LGAs have a Primary Health Care (PHC) Unit, which is headed by a Medical Officer of Health. The unit coordinates PHC activities provided through a number of PHC facilities in the LGAs. In addition, each LGA has one or two secondary healthcare facilities (general hospitals). The PHC centres are manned by a team of health workers comprising of the head (a registered nurse/midwife), four community extension health workers (CHEWs) and five health assistants. Health posts, where available, are manned by junior CHEWs who also assist the community health volunteers/traditional birth attendants (TBAs) where available. Community volunteers are individuals or groups in the community who willingly offer service(s) or undertake a task for the benefit of their community without being paid. These include laypersons, retirees, youths and other community-oriented resources persons (CORPs).

### Selection of participants

A total of 12 focus group discussions (FGDs) were conducted in the study sites among adult male and female community members including adolescents, those aged 30 years and above, and young male and female community members aged between 18 and 29 years. Participants were categorised into literate and illiterate. A total of 73 key informant interviews (KIIs) were conducted. The participants included programme managers, community/local leaders, volunteers, healthcare providers and non-governmental organisation/development partners such as UNICEF. They were identified by snow ball sampling based on their portfolio, and relevance to the introduction of the CDI approach.

### Data collection

Data was collected via FGD and KII techniques. In terms of the FGDs, each session was conducted by a trained moderator and a recorder, and facilitated by a social scientist or an epidemiologist (ASJ and IOA, respectively) on the team. The FGD sessions were conducted in the local language of the studied population in convenient and accessible locations identified by the community – churches, mosques, community halls and PHC centre premises. In terms of the KIIs, interviews took place at the venue approved by the participants such as health facilities, compounds of dwelling places, the traditional head’s place and work places, such as ministries and development agency/NGO offices. The responses were recorded electronically and manually with the permission of the participants. Each session was audio recorded and transcribed. Field notes were collected to supplement the transcripts. Each session lasted between 30 and 60 minutes. An interview guide was used for data collection (see Additional file 2). The guide was based on a structural functionalist perspective gearing towards extracting information on the governance structure and its role in healthcare delivery in the study communities. Factors that have been found in other studies to facilitate community-directed activities and behaviour of people to community participation and community ownership were explored. The issues covered included community governance structure, local and cultural resources for healthcare delivery and cultural perception of health. In addition, existing interventions, community participation, and involvement and challenges of existing interventions were explored. The knowledge of these factors was expected to provide information on potential resources available in the community to support application of a CDI strategy to improve delivery of health interventions. It would also help to understand the layers of relationships in the administration of the communities and how this has impacted on development projects. In addition to the FGDs and KIIs, document reviews at different levels of the health system and at the community level were carried out. To collect the necessary data, a total of eight (8) sets of research instruments developed and harmonised for the participating African countries were used. The information required from the documents reviewed was extracted using a structured checklist.

### Data analysis

The audio-recorded interviews were transcribed in Yoruba, the language which was used to conduct the interviews and FGDs. Research assistants translated the transcripts into English, and translation back into Yoruba was carried out to check for correctness of the translation. Two of the research assistants and two of the authors, a social scientist (ASJ) and an epidemiologist (IOA) listened to the recordings and checked the accuracy of the transcripts and translation. Content analysis was performed independently by the two authors. They analysed the transcripts and field notes for major issues and emerging themes. The responses from different informants or FGD sessions were grouped together, coded and analysed according to themes, and where there was incongruence between the two authors, they re-read the transcripts and made necessary corrections. Both inductive and deductive methods were used to examine the stakeholders’ perceptions – deductive reasoning was employed to draw conclusions from a combination of introduced and emerged themes. The findings were reported in narratives according to the themes and supported by relevant quotations.

## Results

### Prevalent diseases in the communities

Certain diseases are common to certain communities. Identification of these diseases is critical to determine which one to target for intervention and thereby contribute to the CDI process. In the studied communities, several diseases were mentioned as being prevalent in the study communities. These included malaria, fever, stress, typhoid (presumptively diagnosed), measles, convulsion, peptic ulcer, hypertension, diabetes, eye problems, diarrhoea, asthma, cough, tuberculosis (TB), malnutrition, helminthiasis, epilepsy, schistosomiasis and stroke (see Table 2). These diseases were determined based on the illness symptomatology at the community level. The community had nomenclature for some of these diseases which have been published in the literature – and for some diseases such as diabetes, TB, hypertension, fever, typhoid, malaria, asthma, cough and helminthiasis, they used descriptions which fit into medical diagnoses. For diseases such as diabetes and hypertension, apart from the symptomatology, respondents stated that their doctors told them they have the disease. Table 3 shows the community symptomatology of the common ailments. Among the six most cited diseases, malaria ranked first, followed by fever, diarrhoea, cough, typhoid and hypertension.

**Table 2:** Diseases respondents perceived to be prevalent in the study communities

**Diseases**	**Akinyele**		**Ibadan north**		**Ona-ara**		**Ibadan south-west**		**Total frequency of mention**
	**Ojoo**	**Moniya**	**Ala- gbafo**	**Ina- lende**	**Olun loyo**	**Olorun sogo**	**Odo ona/ apata**	**Foko**	
Malaria	X	X	X	X	X	X	X	X	35
Typhoid	X	X	X	X	X	X	X	X	14
Hypertension	X	-	X	X	-	X	X	-	7
Fever	X	-	X	-	X	X	X	-	7
Diarrhea	-	-	X	-	X	X	X	-	6
TB	-	-	X	X	X	-	-	X	4
Cholera	-	X	-	-	-	X	-	X	3
Measles	-	-	-	X	-	X	-	X	3
Eye problem	X	-	X	-	X	-	-	-	3
Diabetes	-	X	-	X	-	-	X	-	3
Convulsion	X	-	-	-	X	-	-	-	2
Cough	-	-	X	-	-	X	-	-	2
Epilepsy	-	-	X	-	-	-	-	X	2
Psychiatry	-	-	X	-	-	-	-	X	2
Ulcer	-	X	-	-	-	-	-	-	1
Asthma	-	-	-	-	-	-	X	-	1
Stroke	-	-	-	-	X	-	-	-	1

X: Indicates communities that mentioned the diseases.

-: Disease not mentioned.

**Table 3:** Community symptomatology of common ailments respondents mentioned are prevalent in the community

**Diseases**	**Local name**	**Local diagnosis/symptomatology**
Malaria/fever	*Iba*	Head ache, high body temperature, loss of appetite, weakness
Typhoid	*Iba Jefun Jefun*	Head ache, stomach pain, loss of appetite, high body temperature, diarrhea
Jaudice related diseases/hepatitis	*Iba ponju poto/ Iba Jedo jedo*	Fever, body weakness, yellow eyes, yellow urine,
Hypertension	*Eje ruru*	Weakness, sleeplessness, head ache
Diarrhea	*Igbe gbuuru*	Watery stool, weakness, dryness of the skin, frequent stooling
TB	*Iko ife*	Cough, weakness, loss of weight, coughing bloody sputum, excessive night sweats
Cholera	*Arun onigbameji*	Watery stool and vomiting at the same time, weakness, dryness of the skin
Measles	*Ita/Olode*	High body temperature, body rashes, loss of appetite in children
Convulsion	*Giri*	High body temperature, inability to eat, weakness, seizures/convulsion
Diabetes mellitus	*Atogbe*	Sugary urine, excessive urination, excessive thirst, weight loss,
Schistosomiasis	*Atosi Aja*	Terminal haematuria
Peptic ulcer	*Ogbe inu*	Epigastric tenderness, peppery epigastric pain, Pain related to fasting,
Asthma	*Iko gule gule*	Cough, difficulty with breathing, chest over expansion during breathing,
Stroke	*Egba*	Weakness, sleeplessness, immobilization, loss of memory

Documents reviewed at the health facilities in the communities revealed that malaria was the most prevalent disease among all age groups and constituted 60–95% of diseases. This was followed by cough (40–50%) and road accidents (30–40%). Apart from malaria, diarrhoea and measles were the common diseases among children under five years of age, while typhoid was mentioned as only being prevalent among adults. Other documented diseases/health problems included helminthiasis and schistosomiasis in children, and maternal-related problems such as complications in pregnancy and death in adult females. According to programme officers at the state and local government levels and the Medical Officers of Health, malaria (mostly), diarrhoea, upper respiratory tract infections and maternity cases were identified as the major health issues among under-served urban populations.

The prevalent diseases identified by the community members and health workers fitted into the pattern of disease in the country as shown by annual reports and other documents reviewed, as well as interviews with key personnel at the national level. In children, communicable diseases – predominantly malaria, upper respiratory tract infection, diarrhoea and measles – were documented as prevalent diseases. In adults, non-communicable diseases (NCDs) – mainly hypertension and diabetes – topped the list.

Of these diseases, malaria was most commonly mentioned as the priority health problem to be addressed. This was corroborated by community members as indicated by a community-based volunteer (CBV) who stated that:

The *most common disease experienced in this community is malaria. Once in a while, maternal mortality also occurs. The reason I said malaria is the most common is because it affects both the young and the old. The other diseases I am aware of, although these are not common, are measles and chickenpox. There are times when chickenpox affects even adults*.

Similarly, a youth leader commented:

…in this type of community, the most common disease is malaria. From my own experience, fever is also common among children especially during the dry season. There are sanitation-related diseases such as diarrhoea, though these are not rampant.

This comment was corroborated by health workers as indicated by a medical officer in Ibadan SW:

T*he most common diseases are malaria, upper respiratory tract infections and pneumonia. Also, due to a high-population density and poor conditions of living, diarrhoea is also common*.

### Social determinants of health

Social factors have been identified as major determinants of health and illness. In this study, the communities’ poverty status, and the gender, cultural beliefs, age, marital status, occupation and social networking skills of its members were identified as the social variables influencing their health status. As according to them, the participants did not have enough funds to utilise health care and desired free health services. In addition, they were mostly of low socio-economic status. The groups affected mostly by the prevalent diseases were children and women. Some of the people who were sick in the community believed that their sicknesses were caused by supernatural forces and therefore resolved not to visit a formal health facility for treatment. For instance, in Alagbafo, a member of the community suggested that we visit a retired hospital worker who refused to go to the hospital for treatment of a chronic skin problem. The patient believed that the illness was of supernatural cause and, hence, was not a case for orthodox medicine.

It was found that the location of the health facility was also a determinant in the utilisation of health services. Many respondents complained that the existing health facilities were far away from where they lived, hence, they went to alternative care providers nearer to them. Most of the respondents identified the cost of treatment and the repeated attacks of some of the diseases, notably malaria, as tapping heavily on their out-of-pocket expenditure and thus increasing the level of poverty in the communities.

In addition, environmental sanitation and water supply were identified as factors contributing to people’s health status. Most of the communities had poor sanitation and water supply. Unlike the urban rich areas which are well connected with good road networks, and are provided with electricity, water and communication facilities, the available infrastructures in the urban poor areas are over stretched due to high-population density and the poor quality of services. They lamented the inability of the government to address these important social and health issues in their communities.

### Healthcare delivery and financing

#### ***Healthcare services***

Primary health care (PHC) is at the cornerstone of the health system and is expected to reach the grassroots level in the LGAs. Access to health services in the study area was limited. As indicated by the respondents, the health facilities were not offering adequate care for most of the diseases prevalent in the community. According to a leader in the Alagbado community in the Ibadan North LGA:

*…the only PHC in the ward is a maternity centre which focuses more on care of pregnant women and some childhood diseases mainly malaria and immunisation, while other age groups and diseases are not adequately covered*.

In the Akinyele LGA, a staff nurse in charge of a health centre lamented the problem associated with healthcare delivery in the community. According to her:

There is no doctor in this health centre. We have requested doctors to visit the health centre regularly, but haven’t had any success. In fact, I am the only nurse here. There are some problems I cannot handle. The people have to go to University College Hospital (UCH), a tertiary facility which is quite a distance from here.

Long waiting times at the clinic were a major concern for community members and a key factor influencing decision on (i) whether to seek healthcare services or treat at home and (ii) the type of health facility/provider to seek. Some quotes from the FGDs and the KIIs support this. According to a male youth leader in Ago-Taylor in the Ibadan Southeast LGA:

…most people go to the health centre because the general hospital (Adeoyo Hospital) is very far from here. Those who can afford private hospital care utilise private clinics, otherwise they treat themselves at home. We are beggars without choice.

A female youth leader in the Akinyele LGA said:

There is no general hospital around. The nearest one is Adeoyo and it is far from here. Many people don’t want to go there because of cost of the transportation and services. People treat themselves at home or go to herbalists. Only very few people go to private hospitals as they are very expensive.

This view was corroborated by all FGD participants in all the study communities. To illustrate the quality of healthcare delivery in the study communities, consider this quote from a male adult FGD participant in Foko in the Ibadan SE LGA:

Healthcare delivery in this community is not good even though there is a health centre. Many people are not happy about the way they are treated because they have to wait for a long time before being attended to. The government should do something about this. The problem is that there are too few nurses for this community. That is why people go to traditional healers or spiritual healers.

Costs of healthcare services may be direct or indirect. Direct costs relate specifically to services such as diagnostics and treatment, and indirect costs relate to transport, food and time needed whilst undertaking health-seeking actions. Generally, healthcare delivery is operated on a fee-for-service basis except for children and women who get some services free of charge. Most of the informants felt that the cost of healthcare services was high and they wanted the government to provide free healthcare services for all. According to a female adult FGD participant in Foko in the Ibadan SE LGA:

……it is too expensive to receive healthcare services in this area. Apart from the fact that drugs are not usually available at the health centre, people spend a lot of money going to Patent Medicine Vendors (PMVs).

This view was corroborated by a PMV who was also a volunteer in a past project in the community:

Patent medicine vendors are the main source of healthcare service delivery in this community. Many people go to PMVs because medicines are not readily available at the health centres and if available, they are costlier because patients have to pay for other services such as a consultation when they go to the hospital.

#### ***Healthcare financing***

According to respondents, health care was financed mainly by out-of-pocket expenses. However to support those who may not be able to afford payments when required, communities translated the existing socio-cultural practices such as the *esusu* (traditional cooperative system) and social groups/CBOs activities to enhance social security and to support members needing assistance. Some of these CBOs are gender defined. It was revealed that females are more into cooperative systems in the study areas. For instance, a female youth leader in the Oke-Itunu area in the Ibadan North LGA indicated that:

….there are women and men associations and clubs. Only very few clubs are a mix of both male and female. We contribute money to help one another in times of need. People take loans from the club and repay over one year. This is helpful.

### Referral services

For the success and efficient implementation of the CDI process, availability of a higher-level health care to attend to complications or cases that cannot be addressed at the community level is essential. It is also beneficial for the referring community healthcare provider to have feedback on cases that may have been referred as a form of a learning experience and information dissemination. The referral system in the Nigerian health system is supposed to be two-way and practiced by all levels of care including alternative care providers. Data revealed that a one-way referral approach was being practiced within the healthcare system. While the health centres referred to secondary health facilities, they did not receive feedback. According to a nurse at a health centre frequented by Inalende residents:

….when we refer patients to Adeoyo (general hospital) we do not receive any feedback whether the patient is doing well or not. The general hospitals do not refer patients back to us for necessary follow-ups or discharge. We are the ones that refer to them without due acknowledgment or feedback.

It was observed that there was no formal arrangement between orthodox and alternative medicine for referral service in the communities. As a result, patients are not referred from one to the other. However, people shuttle between the healthcare providers starting with alternative care delivery services and ending up with the orthodox. According to the Landlord Association chairman in Alagbafo in the Ibadan North LGA:

There are many terrible diseases in this community, such as mental illness. Some people go to traditional healers but when that does not work, they will go to the hospital. Some people even go to church for spiritual healing.

The major problems participants attributed to poor referral service in the areas included poor transport system, poverty, logistics, poor communication among the health facilities, lack of personnel and attitudes of health workers.

### Health interventions in the communities and their delivery mechanisms

#### ***Existing interventions***

The existence of interventions in the community and their success is an indication that such a community may be amenable to intervention delivered by the CDI process. The existing health interventions in the communities include malaria prevention using Insecticide Treated Nets (ITNs) and Intermittent Preventive Treatment for prevention of malaria in pregnancy (IPTp), which are health-facility based. In addition, HIV screening was carried out in the communities by NGOs at designated private health facilities. Participants pointed to the fact that these interventions were mainly for women and children. For instance, an adult male participant in Alagbafo in the Ibadan North LGA argued that:

All the health programmes in this community are designed for women and children. Is there nothing that can be done for adult males and females? If we are sick and not treated, we will transfer the sickness to the children.

Similarly, a female community leader in Olorunsogo in the Ona Ara LGA submitted that:

….children and women are the biggest beneficiaries of health programmes in this community. For instance, the malaria programme was mainly for children. There has not been any health programme in general for adults here.

Documents reviewed at the local government (LG) and community levels showed that immunisation, nutrition, reproductive health, TB and leprosy, environmental health, malaria and HIV/AIDs control programmes were the ongoing interventions in the study areas. Children under five benefitted from immunisation and nutrition programmes, while pregnant women benefitted from the malaria prevention, nutrition, immunisation, reproductive health and HIV/AIDS programmes. All members of the communities benefited from the HIV/AIDS, malaria, nutrition and environmental health programmes. Leprosy and TB programmes were benefited by patients with those diseases. Some organisations also carried out health education on bird flu and malaria.

A woman leader in Foko in the Ibadan SW LGA indicated during a KII that:

….the health intervention I am involved in is immunisation. They (NGOs) usually call us when they want to start immunisation. It is for children and women.

However, some communities mentioned they had no existing health interventions. This was supported in a FGD among male and female residents in Inalende in Ibadan North, where participants mentioned that:

There is no ongoing/existing health intervention in our area. Those politicians are just eating our money without giving us any support like people living in Bodija or other government reserved areas (GRAs) in Ibadan are getting. Please, the suffering is too much…

Provision of policy on health interventions and establishing operational guidelines is a step towards quality assurance and a mechanism to ensure actual delivery of health interventions as intended. Document review at the national level revealed that there were policy documents on malaria, HIV/AIDS, TB and leprosy control, as well as immunisation, environmental health and nutrition programmes. Similarly, as observed at the State Ministry of Health, there were implementation guidelines for all these programmes. According to the manager of the nutrition programme at the Federal Ministry of Health:

Policies are available for Vitamin A, zinc and folic acid interventions. A specified amount was budgeted for the ongoing interventions. The Federal Ministry partners with faith-based organisations (FBOs) and NGOs to carry out some of their activities particularly ones on breastfeeding. However, not much has been done in the urban areas; the emphasis has been on the rural areas. A major weakness is the lack of political will and availability of funds. Support from international organisations, such as UNICEF, has also helped the nutrition unit.

In terms of financial resources, little information was gathered on the funding of existing or past interventions in the communities. No funding was made available for interventions at the community level. At the LG level, it was noted from records at the PHC department that only two of the four local governments had budgets for the existing interventions. At the national level, the financial records were not made available. However, programme officers mentioned that only a proportion of the allocated budget was made available for programmes that implemented the interventions, but these funds were not released on time for effective implementation.

### Mechanisms for delivering existing health interventions

Understanding the delivery mechanisms for existing or recent past health interventions stands to provide evidence for possible implementation of the CDI process. This is with the assumption that the delivery mechanism may be adapted for it. In these study communities, the immunisation programme was being delivered using the house-to-house strategy and was done periodically based on the schedule of the National Program on Immunization (NPI). Children were also reached out to in the markets, religious and social gatherings, schools and playgrounds to ensure wide coverage. Some other interventions, especially those that require health education and training, were carried out in meeting places such as town halls, health facilities, work places, markets and other conducive meeting places depending on the nature of the intervention. Healthcare services were carried out in public premises in the LGAs. For instance, HIV/AIDS control activities such as screening and counselling were carried out in mosques and churches, as well as in schools. Health talks by the NGOs and community meetings, such as socio-cultural and professional association meetings, were also delivered at designated public locations such as markets, churches or mosques. Public awareness campaigns were also carried out in public meetings or gatherings such as statutory community meetings, during worship in mosques and churches, and by town announcers (who walk round the communities announcing messages and/or move around in motor vehicles that have an attached public address system to make announcements).

Home-based strategies have also been used in the study LGAs. The head of a PHC in one of the communities (a senior nurse) mentioned that in the proposed intervention for malaria control under the Integrated Management of Childhood Illnesses (IMCI) strategy, they had trained selected members of the community to be able to treat uncomplicated malaria at home. Similarly, TBAs had been trained to take delivery in the community. She favoured home-based or an approach near homes for delivery of interventions. In addition, she thought that health interventions could also be successfully delivered in faith-based clinics where people could be reached easily and the services provided free or at a subsidised fee.

Another community leader was also supportive of house-to-house delivery of interventions, but not in favour of use of religious settings because not everyone attended these. A youth leader mentioned during a KII that household heads should be used to deliver information and interventions as much as possible so that members do not have to go outside of the community to seek health care. He did not favour the use of markets because people might find these too open and the marketers might find it intruding. According to the director of a PHC centre, the delivery mechanism would depend on the type of intervention. He favoured a community-based delivery of intervention because most of the diseases are preventable and should be tackled at the community level.

In an interview with the State Malaria Control Program Manager, he enumerated the problems with delivery of health interventions and mentioned that they needed to be addressed before planning delivery. The challenges included poor planning, inadequate demographic statistics to assist with planning and attitudes of the health workers.

### Community involvement in projects and health interventions

#### ***Community participation***

Community participation and ownership is critical to the sustainability of community-based interventions. Generally, community participation and its evolution into empowerment have been viewed as a key component to improve health, especially of the poor and disadvantaged. There was evidence that members of the communities participated in community projects. Data revealed that in some communities, members had received a form of training for community projects. A female youth FGD participant in Oke-Itunu in the Ibadan North LGA said:

….people are selected in the community and trained on the specific project being implemented.

The people trained tended to be females and this was related to the type of projects implemented in the communities, which, most of the time, were related to maternal and child health care. Trainees were mostly selected through professional associations and youth organisations. For instance, a male youth FGD participant in the Akinyele LGA revealed that:

….some NGOs came to train us on how to avoid HIV/AIDS. The training focused on the use of condoms and the different ways to contract the disease. We were also trained on how to talk to our friends so that they too can abstain from behaviours that encourage transmission of the disease.

At the community level, a male youth FGD participant in Foko revealed that:

….the research organisations always meet us as a community through the Baale who mobilises the various groups of people concerned to meet and discuss the matter. This gives different groups of people an opportunity to have a say.

Financial contribution by the community is another major form of community participation. Data revealed that some communities had contributed towards development projects in the past. For instance, according to a community leader in Inalende (Chief Imam):

We contributed money to ensure that electricity in this community is functioning well. We also contributed money to build a hospital for which the government promised to supply equipment, but up until now, nothing has been done.

This view was corroborated by a male youth leader in Alagbafo who said:

….people are ready to contribute money if they are sure of the sincerity of the government. As you (the research team) are here now, if the people are sure that you are not deceiving them, they will contribute. People contributed money when the health centre in the community was being built.

The story was different in Oke-Itunu where respondents revealed that they had not contributed money to support a project. According to a female youth leader:

….well, it is strange here because there has not been any health project that brings people together to contribute money. People contribute to repair their gutter and pay for security related activities. But beyond that I am not sure of any health project that people contribute towards. This does not mean that it cannot happen.

#### ***Community engagement***

The stakeholders identified at the community level included the Community Development Association (CDA), the Committee Development Committee (CDC), the Landlord/Landlady Association (LA), and other community-based social organisations such as youth and women’s groups, market women groups, youth organisations, professional associations and cultural groups. The landlord associations and CDCs are gatekeepers in the community, and are relevant for decision making.

**Figure 1:**
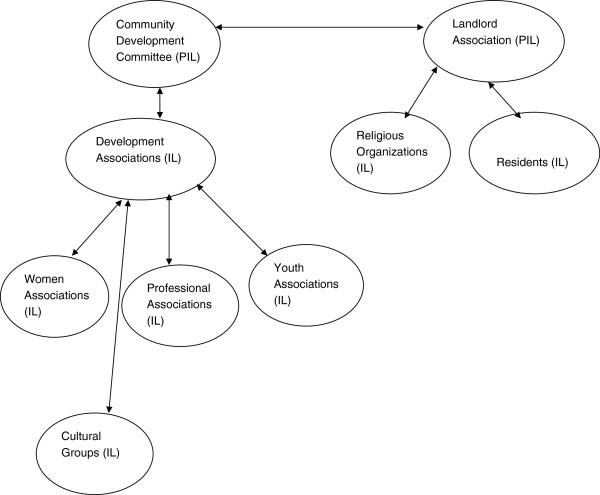
**Stakeholders involved in development projects in the study communities.** Key: P = Power, I = Interest, L = Legitimacy.

Data revealed that both the CDC and LA had obligations to coordinate development activities at the community level. This is because the CDC has formal ties with the LGA while the Landlord Association derives its power from the Residents Association. The interaction among the different stakeholders in the community is shown in Figure 1.

#### ***Public-private partnership***

The public-private partnership is another avenue for service delivery in the study area. Document review at the national level revealed that there was policy on the public-private partnership for HIV/AIDS and malaria control programmes. Public-private partnership policy is included in the country’s 2010–2015 strategic health development plan. However, this is presently being implemented for HIV/AIDS control. The HIV/AIDS control private partners include major companies in Nigeria specifically petroleum, construction and tobacco companies. All these private sectors are under the umbrella of a network called the Nigeria Business Coalition against AIDS (NIBUCAA), and they are involved in the delivery of control interventions.

In the four LGAs, there were numerous private hospitals providing primary and secondary healthcare services of varied quality and at a fee that is unaffordable to most of the residents. In addition to government-funded health facilities, private hospitals and clinics had participated actively in the delivery of some health interventions in the LGAs. Such interventions included distribution of a Vitamin A supplement, family planning, HIV screening and routine immunisation. According to a Director of Public Health at the state level:

Private health institutions are being encouraged to get involved to deliver certain interventions. Some of them have responded positively but this is not yet encouraging.

For example, national immunisation days are carried out in all four LGAs in line with the National Program on Immunization schedule. This programme is supported mainly by multinational donor agencies such as Rotary International, UNICEF, WHO and partly by the Federal Government. These donor-driven interventions are preferably carried out at the community level to ensure access. In some cases, community participation is encouraged through recruitment of community members to deliver such interventions.

Collaboration with stakeholders and with the public health system is another public-private partnership modality for implementing intervention. The activities of NGOs were being carried out in collaboration with PHCs in the Ona Ara LGA, Ibadan SW and Ibadan North LGAs. In the Ona Ara LGA, the Family Health Population Action Committee (FAHPAC) was providing intervention on prevention and treatment of HIV/AIDS. Health education seminars were carried out periodically for groups, mostly youths, and HIV testing was offered to interested members in the community. In the Ibadan SW LGA, Hope Worldwide, an NGO supported by UNICEF and USAID, provided health education talks on HIV/AIDS prevention and awareness campaigns on its treatment to market women and commercial sex workers. In Ibadan North, another NGO, the Primary Health for Maternal Care (PriHMac) carried out a community-based maternal morbidity and mortality reduction programme. Volunteers were trained to identify pregnant women in the community and encourage them to attend a health facility for antenatal care (ANC). They also monitored pregnant women’s compliance to ANC follow-up visits, use of drugs and immunisation. The programme also empowered the health facilities to support ANC, provide free malaria testing kits and conduct training for midwives in the health facilities.

#### ***Minimum intervention package to be delivered with the new strategy***

Stakeholders were engaged in what they considered should be the minimum intervention package to be delivered with the new CDI strategy. Treatment of malaria topped the list of their agenda. Almost all the respondents in the study believed that any new intervention strategy should focus on malaria.

Another important suggestion for a minimum intervention package was environmental sanitation. There was consensus that this was a major problem facing the studied communities. For them, improving environmental sanitation would enhance the health of the people. Respondents narrated their efforts to make their environment clean but stated that they needed support from the government. According to an adult male participant in a FGD:

….we want the government to provide facilities for regular collection of waste so that the environment will be free of bad odour. Most of the time, there’s dirt everywhere. This is not good for our health.

Respondents also discussed the state of their water supply and concluded that it was a major source of worry. There was consensus among KII and FGD participants to request a pipe borne water or a borehole. This was corroborated by an NGO programme officer who explained that:

….provision of intervention for issues other than for diseases such as water supply and waste disposal will go a long way to improving the health conditions of the people as most of the communities lack basic social amenities.

Generally, chairmen of landlord associations lamented the state of neglect of their areas and called on the government to pay attention to the needs of the people.

Free healthcare delivery is another package suggested by the participants. It was generally agreed that free medical service will alleviate the suffering of people as the National Health Insurance Scheme (NHIS) is not yet available to everybody. This was supported by quotes such as:

….unless the government makes treatments free, it will be difficult for many people to access treatments because many people are poor.

Many women go to faith healers now not because they do not know that it is better to have treatment in the hospital, but because they cannot afford it. Many of our women are not employed and some of their husbands have lost their jobs.

Apart from the suggested interventions for a minimum package, some programme managers opined that if a volunteer was to be engaged in more than one intervention, the number of interventions should not be more than two, and at most three, depending on the demand of the interventions. This was based on their experience working with some of the volunteers, their perception of the capabilities of the volunteers and their level of education, as well as the fact that the volunteers have to do their personal daily chores to generate income.

### Resources available in the communities for CDI process

The resources available in the communities are shown in Table 4. These have been grouped into human, financial and infrastructure/support resources, and are presented according to their availability in each of the communities studied.

**Table 4:** Potential resources available for community participation in CDI process

	**Resources**	**Akinyele**		**Ibadan north**		**Ona-ara**		**Ibadan south west**		**Potential roles of available resources**
		**Ojoo**	**Moniya**	**Ala Gbafo**	**Ina lende**	**Olun loyo**	**Olorun sogo**	**Odo-ona/Apata**	**Foko**	
Human resources	Project monitoring committee (Vigilante)	x	x	x	x	x	x	x	x	Project monitoring
	Social audit	x	x	x	x	x	x	x	x	Project monitoring and evaluation
	Community Development Committee/Landlord Association/Professional associations	x	x	x	x	x	x	x	x	Community entry and mobilization
	Traditional Birth Attendants (TBAs)	x	x	x	x	x	x	x	x	Volunteering service
	Community Volunteers	x	x	x	x	x	x	x	x	Volunteering service
	Role Model Caregivers (RMC) for Home Management of Malaria [not yet functioning]	x	x	x	x	x	x	x	x	Volunteering service
	Patent Medicine Sellers	x	x	x	x	x	x	x	x	Volunteering service
	NGOs/Development Partners/FBO/CSO	x	x	x	x	x	x	x	x	Partnership
	Past training of volunteers for Intervention projects	x	x	x	x	x	x	x	x	Skill Reservoir
Financial resources	*“Esusu”* (Traditional cooperative system)	x	x	x	x	x	x	x	x	Financial resource
	Monthly contribution for security	x	x	x	x	x	x	x	x	Financial resource
	Provision of incentive to volunteers: All provided were non-monetary e.g. gifts, exemption form communal labour and recognition at annual meetings	Nil	Nil	x	Nil	Nil	Nil	Nil	x	Motivation and recognition
	Financial participation in existing health interventions	No funding was made available to existing health interventions at the community level. However a community mentioned they contributed money for building of a health center in the past								Community participation and ownership
Infrastructure/support resources	Community Records: Record keeping at community level	Nil	Nil	x	x	Nil	x	x	x	Record keeping
	Volunteer Records: Record keeping by volunteers	NA	NA	x	NA	x	x	x	x	Record keeping
	Community Hall/Meeting Place	x	x	x	x	x	x	x	x	Meeting venue
	Worship places	x	x	x	x	x	x	x	x	Community mobilization
	Schools	x	x	x	x	x	x	x	x	Community mobilization
	Transportation – Motor cycle	x	x	x	x	x	x	x	x	Transportation and volunteering
	Market places	x	x	x	x	x	x	x	x	Community mobilization

Notably among the human resources at the community level are the community development committees, the landlord/landlady associations and volunteers. Community volunteers existed in all of the study communities. These include village health workers, traditional birth attendants, role model caregivers for home management of malaria (RMC) and community-based medicine distributors. The names of some of these volunteers are used interchangeably. While supporting the use of community volunteers, the Director of Nutrition at the national level thought that:

Underserved people are involved in health service delivery by using community members to implement growth monitoring. These community members are selected by the communities. The volunteers are not enough but there are no incentives so no one else is interested in the job either.

According to the state Malaria Control Programme Officer, all LGAs have volunteers who have been trained as role model caregivers for home management of malaria as far back as 2010, however, they have not been functioning because the scale up of the HMM strategy in Nigeria has been delayed. Some of these volunteers are recognised by the Local Government Authority, although there is disenchantment as they are not compensated and are not provided incentives. A few of the volunteers mentioned that they were motivated by gifts in recognition of their services at annual programmes/meetings, success of their contribution to the health of the community, training received on assignment and occasional exception from community dues.

The KII and FGD participants believed that incentive was important for mobilising people to volunteer. However, there was divergent opinion about the modalities for providing incentives. These included whether it should be financial, community recognition and/or capacity building.

At the health system level, there was a dearth of quality, quantity and mix of healthcare workers with a skewed distribution towards an over-served urban area. The PHCs in the study areas were manned by Medical Officers of Health, matrons, nurses/midwives, CHEWs, CHOs and health attendants. However, in many instances, the health workers were not there for the populace and the quality of service provided was sub-optimal because of poor job aids and infrastructure, such as vehicles that are needed for home visits, and community health education. These, consequently, resulted in the community seeking alternative health care in the private sector, traditional and faith-based healing clinics, and practicing home based/self-treatment.

The NGOs and FBOs constituted important resources available in the community. The former, especially, collaborated among themselves and partnered with the communities. One official of an NGO said:

We collaborate with other NGOs to carry out specific activities. Generally, this collaboration exists because some grants demand that a consortium of NGOs have to come together to tackle a particular problem. So there is a coalition of NGOs even here in Ibadan.

Other potential social, professional and cultural networks that can contribute to the implementation of a CDI strategy for delivery of interventions include the CDCs, landlord associations, professional associations, youth associations, age grade groups, social clubs, religious organisations and women’s associations. For instance, an NGO manager revealed that youths are a major force in community participation:

Actual participation in interventions in this community is strengthened by the support received from the youths. They mobilise for support and motivate their members to participate.

Women leaders were of the view that women had a role to play in service utilisation and they indicated that they constituted a major source of volunteer services in the community. They have committees from which NGOs select volunteers to implement interventions in the community. For example, a woman said that:

….we motivate other women to utilise available or newly-introduced interventions.

It was also revealed that health issues are discussed at women’s forums where important messages are passed across to members. According to a female youth:

There are various community-based women’s associations. Some organise workshops where maternal and child health issues are discussed. Immunisation delivery has been effective using this network of women’s groups.

Data revealed that the CDA and/or the Landlord Association occupied the apex of decision-making in the communities, and as a result, they would be a major port for community entry and advocacy.

Considering the resources available in the LGAs, the LGAs were urban based and had similarities in terms of diversity in their cultures. Even though Yoruba was the major ethnic group, there were also other ethnic groups. However, they were organised in the same way and their governance structure was similar. The CDA and the Landlord Association were the basis of governance in the communities, and social and professional associations were common to all of them all (see Figure 1). The LGAs were different in the sense that Ibadan North and Ibadan SW LGAs have secondary and tertiary health facilities unlike Akinyele and Ona Ara LGAs where these levels of health care were not present, and thereby the PHC facilities were not optimally functional. The unity in diversity in the study population could be harnessed to achieve cooperation and participation in the CDI process. The people share a common voice for development and they could promote acceptance of intervention within the different groups if adequately informed.

On the level of preparedness of the communities for the CDI project, it was revealed that the existing administrative structures in the communities have been used for development projects in the past. According to the officials of the CDA and the Landlord Association, as well as ethnic associations, whenever there is any project to be executed in the communities, the CDA or the Landlord Association chairpersons would mobilise all other associations and individuals for the project. According to a Landlord Association chairperson in Alagbafo:

When we have a project to execute, we get all the groups in the community together and share the responsibilities. This has helped us a lot in the past. So, if there is another project, as you are proposing, our people are ready.

This view was corroborated by chairpersons of ethnic and professional associations in the study communities. The various available resources in the communities as shown in Table 4 support the fact that most of the communities will be ready for the CDI process. However, the aspect of community record keeping, provision of incentives and financial participation in health-related projects at the community level need to be addressed.

## Discussion

In this study, findings showed that many residences in the core metropolis of Ibadan were characterised by overcrowding, poor hygiene and sanitation, and the absence of proper social amenities and civic services. Most urban communities have been shown to also face various health challenges including communicable diseases, NCDs, maternal and child health problems, natural disasters and threat of re-emerging and emerging diseases. The high prevalence of malaria in these communities, which incidentally corroborates the trend in the country, suggests the inadequacy of the Roll Back Malaria programme to combat the disease in Nigeria. Community-based interventions using the CDI strategy will definitely go a long way to reduce the prevalence of malaria in the country as this study has shown that community-based medicine distributors (CMDs) have a great influence on members of their communities, in terms of mobilising them and encouraging adherence to health interventions.

The inadequate infrastructure and public utilities, as well as poor environmental hygiene, prevailing in this study environment could readily explain the poor health status of the communities and the high prevalence of communicable diseases in the Ibadan metropolis. This finding supports the WHO’s concern about the sub-optimal health of the urban poor which informed the WHO’s request for support towards promoting urban planning for healthy behaviours and safety, improvement of urban living conditions, ensuring participatory urban governance, building inclusive cities that are accessible and making urban areas resilient to emergencies and disasters [7].

This study found that governance in the urban areas is quite different to that which exists in the rural areas. While traditional leaders represent the interest of the rural communities, community development associations/committees (CDAs/CDCs) remain the local governing body and gateway to urban communities. It has been argued that good governance helps to ensure that opportunities and advantages are more evenly distributed, and that access to health care is fair and affordable [7]. In urban areas, governance is often met with different opinions and opposition from various groups because of the diversity of knowledge and cultures that abound. These often lead to more robust contributions that have enabled urban-oriented arrangements and social networks to be translated to development opportunities in different parts of the world [8], including Nigeria.

In traditional African societies, community development is rooted in lineage and communal arrangements usually promoted by consanguineous relationships. However, the diffuseness of the urban setting has necessitated a new arrangement of formal organisations and professional associations [9]. The social networks and organisations in existence for community development in this study include the CDAs, CDCs, landlord associations, professional and cultural associations, youth organisations and women’s groups. Apart from the purpose of maintaining cultural ties and a sense of collective development, people of different ethnicity also tend to affiliate together in urban communities to provide social amenities such as electricity, and security of life and property. In some communities, these development programmes have been extended to support the health care of community members through subsidies or cooperative loans. These social networks and organisations were also identified by Mohammed, Idowu and Kuyinu and in their study, they suggested that there is a need to improve community participation [10]. Judging from their current role in community development and implementation of existing health interventions, the CDAs, landlord associations and the various other associations can be mobilised to contribute to the implementation of the CDI process.

In addition to the CDAs, there is multiplicity of civil society organisations (CSOs) in the form of non-government organisations (NGOs) and faith-based organisations (FBOs). These operate in different capacities connecting the communities to government and international development partners, as advocates for community development and good healthcare services, custodians of human rights, and promoters of good morals and health. There was a general consensus that NGOs engage in advocacy and support implementation of programmes at the state and local government levels. The NGOs are mostly involved in empowerment programmes and primary health care (PHC) delivery, providing such services as safe motherhood, HIV education and referral. They conduct health education on various health issues, and train volunteers such as peer educators, change agents and implementers of interventions. Some NGOs work with commercial sex workers and train some of them as peer educators. They provide health education on safe sex and train them to have vocational skills with the ultimate aim of encouraging them to change to another occupation. Hence, the NGOs could assist in the implementation of the CDI programmes through their close working relationships with the informal organisations and community volunteers.

Collaboration between development agencies such as UNICEF, UNAIDS, WHO, and the NGOs, FBOs and other CSOs in the urban areas has been a major source of development in modern-day society [11]. This study revealed that there was collaboration among the various NGOs working in the communities as development agencies work through these NGOs to deliver interventions. Therefore, this collaboration, if properly utilised, can strengthen the CDI process. The public-private partnership is another process of service delivery in the study area. The established public-private partnership for HIV/AIDS and malaria control programmes in these communities can be harnessed for the CDI strategy.

This study found that urban under-served populations have limited access to healthcare services. There is a disparity in the distribution of health facilities in the LGAs. While the urban rich areas have access to modern healthcare services as they are served with secondary and tertiary institutions, the urban poor areas have limited and unequal access. The primary healthcare facilities that are supposed to serve them are ill equipped or non-existent. Although private and alternative sources of healthcare delivery exist, economically, most of the indigenous residents are not buoyant, hence, the fees charged were found to be unaffordable, and this precluded the residents’ utilisation of health facilities. Most of the informants in this study felt that the cost of healthcare services was high and they wanted the government to provide free healthcare services for all. Fortunately, the residents in these urban poor areas have translated the existing socio-cultural practices such as the cooperative system to advantage healthcare support. This is an improvement from mid-2012 when only about three percent (3%) of Nigerians, many of whom were in urban areas, had access to the National Health Insurance Scheme (NHIS) [12]. The CDI approach would benefit from co-opting the existing socio-cultural structure into implementation of health interventions.

Delivery mechanisms for existing interventions in the community could provide direction for the implementation of a CDI strategy. Community-based delivery of interventions, such as the house-to-house strategy, and health education at work places and markets, religious gatherings and public meetings were favoured thus supporting the fact that CDI process stand to be acceptable to the urban poor. Some of these interventions were delivered in collaboration with health facilities and NGOs in the communities. With the demonstrated effectiveness of these delivery mechanisms, they stand to be feasible for the CDI process. The reason for preferring community-based delivery of health interventions is the perception that most of the diseases, which are preventable, are sequel to the poor environment and infrastructure in the community. Hence, they should be tackled at the community level.

Findings in this study support the fact that community involvement is critical to the successful implementation of community-directed health interventions. The community role as an advocate, mobiliser, monitor and collaborator was demonstrated in this study. A study in Cuba [13]^,^ which took advantage of the existing community-based organisations in the implementation of community-directed projects, showed that extensive community involvement occurred and that this played an active role in mobilising community members and enhancing linkage systems critical to the project’s success. In addition, women played bigger roles than men in health-related interventions outside their households – a phenomenon similar to findings in this study which found that many of the volunteers in existing interventions were women [13].

Human resources for health are an important issue in the CDI process. This study revealed that community volunteers are the major human resource available in communities. Several authors have demonstrated that community-based healthcare providers which comprise of traditional birth attendants, patent medicine sellers, paramedics, lay mother volunteers, role model caregivers for home management of malaria (RMC) and other community-oriented resources persons (CORPs) have the ability to provide effective community/home-based treatment of malaria, which subsequently reduced morbidity and mortality from the disease [14,15]. There is a growing demand for these groups of care providers to take on not only the management of malaria but also pneumonia, diarrhoea and other appropriate childhood diseases [5]. The community-based healthcare providers identified in this study were engaged in not only malaria control but also programmes designed for the reduction of maternal mortality and malnutrition, as well as for delivering immunisation services. The multi-country study carried out to test whether CDTi could be used to deliver a broader package of interventions alluded to the feasibility of using volunteers for delivering multiple interventions [4]. It also provided evidence that at least four to five interventions could effectively be implemented through CDI strategies. However, opinions gathered in this study suggest that the maximum number of interventions to assign to community-based volunteers should not be more than three. The preference for lower number of interventions may be related to rural–urban differences because the urban volunteers could be busier fending for daily living and may have more challenges in the delivery of the interventions in communities that are densely populated and are of different ethnic groups and culture.

Findings in this study showed that community volunteers constitute a potential resource for the delivery of the CDI process. To maximise the services and time of volunteers/ community-based healthcare providers, especially if the minimum package for healthcare delivery is encompassing more than one intervention, they must be seen to be capable and acceptable. They need to be provided incentives and empowered to be able to cope with the demand of the urban poor setting. In this study, many of the volunteers alluded to receiving training on the specific project they assisted on. However, their financial standing and ability to cope with the demands of volunteering and their routine chores to generate money for their household were not addressed. This calls for developing appropriate selection criteria and mechanisms for providing incentives. One other factor that may threaten the use of community-based healthcare providers/volunteers for the CDI strategy in urban slums is poor recognition of volunteers by the community. Although in this study area the local governments recognised their existence, there was no formal incentive provided and the community had no incentive structure in place. High attrition rates among community-based volunteers, which often resulted from a lack of motivation and incentives, could be a major obstacle to their sustainability and effectiveness in any intervention [5]. This was the finding of a study which used CMDs for distribution of artemisinin-based combination therapy (ACTs) to treat uncomplicated malaria in rural communities in one of this study’s LGA. In this case, many of the trained volunteers dropped out of the assignment because of poor incentives and the community’s relent on pledges to provide support such as financial assistance to pay for their transportation in order to be able to replenish drug stocks, recognition and exemption from communal labour, and financial levies [16].

## Conclusion

The findings in this study support the feasibility of using the CDI process in delivering health interventions in urban poor communities and show that potential resources for the strategy are abound in the communities. The evidence for this submission includes: i) the available potential community resources, especially human resources, some of which are experienced in community volunteering work; ii) the presence of support structures, such as health care facilities, NGOs and CSOs in the community; iii) the existence of CBOs/CDAs and other socio-cultural groups which could facilitate mobilisation of community members to participate actively in health interventions; iv) existing collaboration and linkages between the various stakeholders; v) ongoing community-based health interventions, vi) perception that many of the prevalent diseases can be tackled at the community level and vii) the responsiveness of the local government officials to projects introduced into the LGAs. However, the following weak components were identified: provision of incentives and recognition of volunteers, support by the government to provide infrastructure, basic social amenities and adequately-equipped PHC facilities to support community-based health workers. For the CDI approach to be practiced effectively, these need to be addressed and strengthened before the next steps can be taken.

## Competing interests

The authors declare that they have no competing interests.

## Authors’ contributions

All the authors contributed to the conceptualisation of the study. Data collection was supervised by ASJ, IOA. ASJ and IOA analysed the qualitative data and prepared the draft, and COF contributed to the conceptualisation and implementation, as well as the revision of the draft. JS contributed to the protocol development, supervised analysis and interpretation of the data, and revised the draft. All authors contributed, read and approved to the revision and final draft.

## Supplementary Material

Additional file 1 Multilingual abstracts in the six official working languages of the United Nations. Click here for file

Additional file 2 FGD and KII guides. Click here for file
