# Supplementary material for: Assessing resources for implementing a community directed intervention (CDI) strategy in delivering multiple health interventions in urban poor communities in Southwestern Nigeria: a qualitative study
Source: Infect Dis Poverty. 2013 Oct 24;2:25. doi: 10.1186/2049-9957-2-25 (PMC4177198; doi:10.1186/2049-9957-2-25)
Supplement: Additional file 1 — Multilingual abstracts in the six official working languages of the United Nations. [file 2049-9957-2-25-S1.pdf]

Translation of the abstract into the six official working languages of the United Nations

تقييم الموارد اللازمة لإنشاء استراتيجية تدخل مجتمعية لتوصيل حملات صحية متعددة بالمجتمعات الريفية الفقيرة بجنوب غرب نيجيريا: دراسة نوعية

إكليووابو أو أجايا، أيوديل أس جيجيد، كاثرين أو فالاد، جواهانس سمرفيلد

ملخص

خلفية: تطور العديد من معايير التحكم بالأمراض البسيطة والميسورة التكلفة والفعالة وذلك نظرًا لصعوبة الوصول وخاصة الوصول إلى السكان الأكثر فقرًا (الريف والحضر) والمشاركة الغير ملائمة للمجتمع. أفضل الاستراتيجيات للتعامل مع مشاكل وصول الحملات الصحية هي مبادرات الحملات الصحية الموجه والتي نجحت بالمناطق الريفية. أجريت هذه الدراسة لتقييم الموارد لاستخدام استراتيجية توصيل الحملات المجتمعية الصحية لتوصيل خدمات صحية بالمجتمعات فقيرة الخدمات بمدينة إيبادان بدولة نيجيريا.

المنهج: أجريت دراسة تقويمية بثمانية مجتمعات فقيرة بمدينة إيبادان متروبوليس بوباية أويو. شملت الدراسة 12 مجموعة دراسة للمناقشة مع أعضاء المجتمع و73 مقابلة تقويمية رئيسية مع قادة المجتمع ومديري البرامج والمنظمات المجتمعية والمنظمات الغير حكومية وبعض أصحاب المصالح على المستوى الفيدرالي والحكومي والمحلي استخدمت لتجميع البيانات لتحديد الأمراض المتفشية والخدمات الصحية، بالإضافة إلى اكتشاف الموارد المحتملة لاستراتيجية التدخل المجتمعي لتوصيل الخدمات الصحية. سجلت جميع المقابلات صوتيًا. واستخدم تحليل المحتوى لتحليل البيانات.

النتائج: أسفرت الدراسة عن أن الملاريا وأمراض الجهاز التنفسي والإسهال والحصبة هي الأكثر انتشارًا بين الأطفال، بينما تقدمت أمراض الضغط العالي والسكري بقائمة الأمراض التي يعاني منها الكبار. مول الرعاية الصحية بصفة رئيسية من التبرعات المباشرة. اعتبرت الدراسة التكلفة والموقع من العوائق التي تواجه الحملات الصحية: التعامل غير الرسمي من مؤسسة إسوسو قدم دعمًا لغير القادرين على تكلفة الرعاية.

شملت حملات الرعاية الصحية مواجهة أمراض المناعة والتغذية وطب الأسرة والسل والجذام والصحة البيئية والملاريا والإيدز. تضمنت الحملات توصيل للمنازل ورعاية منزلية وتوعية صحية وحملات. اعتبر ت المشاركة المجتمعية بتخطيط المشاريع وتنفيذها ومراقبة تطورها. تتضمن الموارد المتاحة لهذه النشاطات والتي تمثل الموارد المحتملة لعملية التدخل المجتمعي للحملات الصحية: المتطوعون والمنظمات الغير حكومية والمنظمات المجتمعية أما سوى ذلك فهم ملاك ومحترفون وسيدات ومنظمات شباب ومراكز اجتماعية والمنشآت الصحية المتاحة.

.الخلاصة: تدعم هذه النتائج إمكانية استخدام عملية التدخل المجتمعي للحملات الصحية لتوصيل الحملات الصحية بالمجتمعات الفقيرة الريفية وتظهر الموارد المحتملة لاستراتيجية تستند إلى المجتمع.

Translated from English version into Arabic by Laila Mostafa, through

TRANSLATORS
WITHOUT BORDERS

在尼日利亚西南部城市贫困社区实施提供多种健康干预的社区导向干预（CDI）策略的资源评估：一项定量研究

Ikeoluwapo O. Ajayi, Ayodele S. Jegede, Catherine O. Falade, Johannes Sommerfeld

摘要

引言：由于渠道不畅，特别是难以惠及城乡贫困人口以及社区参与不足，许多简单、经济、有效的疾病控制措施的影响有限。解决卫生干预可及性问题的有效策略是社区导向干预（CDI），该方法在农村地区已成功应用。本研究在尼日利亚伊巴丹健康干预服务欠佳的城市社区进行，对使用 CDI 策略提供健康干预的资源进行评估。

方法：本研究在奥约州伊巴丹的的八个城市贫民社区开展。采用专题小组讨论（FGDs）和访谈（KIIs）对采集到的数据进行定性评价，以确定流行的疾病和医疗卫生服务工作，并探索 CDI 策略的潜在资源。组织社区成员进行了 12 次专题小组讨论，对社区领导、项目经理、社区组织（CBOs）、非政府组织（NGO），以及联邦、州和地方各级政府的其他利益相关者进行了 73 次访谈。所有访谈录制音频。采用内容分析法对数据进行分析。

结果：在儿童中流行的疾病有疟疾、上呼吸道感染、腹泻和麻疹，成人中高血压和糖尿病排名居前。医疗费用主要是患者自付。成本和地理位置被确定为利用卫生设施的障碍。非正式合作团体（或合作社）（*esusu*）可为无力支付治疗费用者提供支持。免疫接种、营养、生殖卫生、结核病和麻风病、环境卫生、疟疾和艾滋病防治等项目正在进行中。提供的防治策略包括挨户的、以家庭为单位的治疗、健康教育和宣传活动。常见的做法有社区参与发展项目的规划、实施和监测。为这些活动提供的资源和构成 CDI 潜在资源的包括社区志愿者、CBOs 和 NGOs。其他的还有业主、专业人士、妇女和青年协会、社交俱乐部、宗教组织和可利用的卫生设施。

结论：本研究结果支持在城市贫困社区 CDI 提供健康干预的可行性，表明在社区潜在的资源比比皆是。

Translated from English version into Chinese by Yang Pin, through

CHINESE CENTER FOR DISEASE CONTROL AND PREVENTION
NATIONAL INSTITUTE FOR PARASITIC DISEASES

Évaluation des ressources pour la mise en place d'une stratégie d'intervention dirigée au niveau communautaire (IDC) dans la réalisation d'interventions sanitaires multiples dans les communautés urbaines défavorisées du sud-ouest du Nigeria : étude qualitative

Ikeoluwapo O. Ajayi, Ayodele S. Jegede, Catherine O. Falade, Johannes Sommerfeld

Résumé

Contexte : De nombreuses mesures simples, peu coûteuses et efficaces de lutte contre les maladies ont pourtant peu d'impact en raison de difficultés d'accès, notamment pour les populations pauvres (urbaines et rurales), et de mauvaise participation des communautés. Utilisée avec succès dans les zones rurales, les interventions dirigées au niveau communautaire (IDC) constituent une stratégie éprouvée pour résoudre le problème de l'accès aux interventions sanitaires. La présente étude a été réalisée dans le but d'évaluer les ressources disponibles pour le déploiement d'une stratégie d'IDCI pour des interventions sanitaires auprès des communautés urbaines mal desservies d'Ibadan au Nigeria.

Méthodologie : Une étude formative a été effectuée dans huit quartiers pauvres de la métropole d'Ibadan, dans l'état d'Oyo. Une approche qualitative, avec 12 discussions de travail avec les membres de la communauté et 73 entretiens avec des informateurs principaux comprenant les chefs des communautés, des responsables de programme, des organisations communautaires, des organisations non gouvernementales et autres parties prenantes au niveau de l'administration fédérale, d'état et locale, a été employée pour recueillir des données afin de déterminer les maladies prévalentes et les services de soins et d'évaluer les ressources potentielles pour une stratégie d'IDC. Tous les entretiens ont été enregistrés. Une analyse des contenus a été réalisée pour analyser les données.

Résultats : Paludisme, infections des voies respiratoires hautes, diarrhée et rougeole s'avèrent les affections prévalentes chez les enfants, tandis que l'hypertension et le diabète arrivent en première place chez les adultes. Les soins de santé sont généralement payés par les patients eux-mêmes. Les obstacles à l'usage des installations sanitaires identifiés sont le coût et la localisation. Les personnes qui n'ont pas les moyens de payer les soins bénéficient du soutien de coopératives informelles (*esusu*). Les interventions en cours comprenaient des programmes de vaccination, de nutrition, de santé reproductive, de lutte contre la tuberculose et la lèpre, de santé environnementale, de lutte contre le paludisme et le VIH. Les stratégies de réalisation

comprenaient des traitements à domicile au porte à porte, une éducation sanitaire et des campagnes. La participation de la communauté à la planification, à la réalisation et au suivi des projets de développement a été décrite comme habituelle. Les ressources disponibles pour ces activités, qui constituent autant de ressources potentielles pour les IDC, comprennent les bénévoles de la communauté, les organisations communautaires et les ONG. On peut également citer les propriétaires des logements, les professionnels, les associations de femmes et de jeunes, les clubs sociaux, les organisations religieuses et les centres de santé existants.

Conclusion : Les résultats de notre étude confirment la faisabilité d'une approche par IDC pour la réalisation d'interventions sanitaires dans les communautés urbaines défavorisées. Ils démontrent que les ressources potentiellement mobilisables pour cette stratégie sont largement présentes dans ces communautés.

Translated from English version into French by Suzanne Assenat, through

TRANSLATORS
WITHOUT BORDERS

Оценка ресурсов для осуществления стратегии интервенций, нацеленных на сообщество (ИНС) при проведении нескольких медицинских интервенций в бедных городских поселениях на юго-западе Нигерии: качественное исследование

Икеолувапо О. Аджайи, Айоделе С. Джегеде, Катрин О. Фаладе,
Йоханнес Зоммерфельд

Реферат

Общие сведения: большое число простых, доступных и эффективных мер по контролю над заболеваниями имели ограниченное воздействие из-за плохого доступа, особенно среди самых бедных групп (городского и сельского) населения, и недостаточного участия сообщества. Подход интервенций, нацеленных на сообщество (ИНС), который успешно используется в сельской местности, − это проверенная стратегия, которая решает проблему доступа к медицинской помощи. Данное исследование было проведено с целью оценки ресурсов для использования стратегии ИНС при оказании медицинской помощи в городских поселениях с низким уровнем медицинского обслуживания в Ибадане, Нигерия.

Методы: в восьми бедных городских сообществах одного из крупнейших городов страны, Ибадане, штат Ойо было проведено формативное оценивание. При сборе данных с целью определения превалирующих заболеваний и медицинских услуг, а также изучения потенциальных ресурсов для стратегии ИСВ были применены качественные методы, которые включали в себя 12 обсуждений в фокус-группах (ОФГ) с членами сообществ, а также 73 интервью с ключевыми информантами (ИКИ), среди которых были руководители сообществ и программ, представители общественных (ОО) и неправительственных организаций (НПО), и другие заинтересованные представители правительства на федеральном и местном уровнях, а также на уровне штата. Была произведена аудиозапись всех интервью. При анализе данных использовался метод контент-анализа.

Результаты: было обнаружено, что малярия, инфекции верхних дыхательных путей, диарея и корь преимущественно распространены среди детей, а гипертония и диабет − среди взрослых. Оказание медицинской помощи финансируется главным образом за свой счет. Стоимость услуг и места их предоставления были определены как

препятствия в использовании медицинского обслуживания. Для тех, кто не в состоянии оплатить стоимость медицинской помощи, существуют неофициальные кооперативы (*эсусу*). Действующие программы медицинских интервенций включали программы по вакцинации, контролю над питанием, охране репродуктивного здоровья, борьбе с туберкулезом и проказой, контролю над гигиеной окружающей среды, а также программы по борьбе с малярией и ВИЧ/СПИД. Способы оказания медицинской помощи включали обход всех домов, лечение на дому, санитарное просвещение, а также кампании по воспитанию сознательного отношения к здоровью и санитарному просвещению. Участие в планировании, осуществлении и мониторинге проектов по развитию представителей сообществ были отмечены в качестве общепринятой практики. Ресурсы, доступные для этих мероприятий, которые представляют собой потенциальные ресурсы для процесса ИСВ, включают добровольцев, действующих от имени сообществ, ОО и НПО. Они также входят землевладельцы, профессиональные, женские и молодежные ассоциации, общественные клубы, религиозные организации и существующие медицинские учреждения.

Вывод: результаты данного исследования подтверждают возможность использовать процесс ИСВ с целью внедрения медицинских интервенций в бедных городских поселениях и свидетельствуют об изобилии потенциальных ресурсов для внедрения упомянутой стратегии в этих поселениях.

Translated from English version into Russian by Halyna Maksymiv, through

TRANSLATORS
WITHOUT BORDERS

Evaluación de recursos para la puesta en marcha de una estrategia de Intervención Directa en la Comunidad (IDC) con el fin de realizar múltiples intervenciones sanitarias en comunidades pobres urbanas del Suroeste de Nigeria: un estudio cualitativo

Ikeoluwapo O. Ajayi, Ayodele S. Jegede, Catherine O. Falade, Johannes Sommerfeld

Resumen

Antecedentes: Muchas medidas sencillas, económicas y efectivas para el control de las enfermedades tienen un impacto limitado debido al difícil acceso, especialmente en las poblaciones más pobres (urbanas y rurales) y a una inadecuada participación en la comunidad. Una estrategia de validez demostrada para solucionar este problema de acceso a las intervenciones sanitarias se basa en las Intervenciones Directas en la Comunidad (IDC), que se han puesto en práctica con éxito en zonas rurales. Este estudio se llevó a cabo para evaluar los recursos para el uso de una estrategia IDC con el fin de realizar intervenciones sanitarias en comunidades urbanas mal atendidas en Ibadan (Nigeria).

Métodos: Se efectuó un estudio formativo en ocho comunidades pobres urbanas dentro del área metropolitana de Ibadan, en el estado de Oyo. Se utilizaron métodos cualitativos que comprendían 12 debates en grupos concentrados (DGC) con miembros de la comunidad y 73 entrevistas con informantes clave (EIC) a líderes de la comunidad, responsables de programas, organizaciones basadas en la comunidad (OBC), organizaciones no gubernamentales (ONG) y otros participantes a nivel del gobierno federal, estatal y municipal, con el fin de recoger datos para determinar las enfermedades predominantes y los servicios sanitarios, así como para explorar los potenciales recursos para una estrategia IDC. Todas las entrevistas fueron grabadas en audio y su contenido se utilizó para analizar los datos.

Resultados: Se llegó a la conclusión de que la malaria, la infección del tracto respiratorio superior, la diarrea y el sarampión eran prevalentes en niños, mientras que hipertensión y diabetes encabezaban la lista de enfermedades entre adultos. La atención sanitaria se financió principalmente mediante gastos de bolsillo. El coste y la ubicación fueron identificados como obstáculos para la utilización de instalaciones sanitarias; había cooperativas informales (*esusu*) disponibles para ayudar a quienes no pudieran pagar sus cuidados. Programas de control de inmunización, nutrición, salud reproductiva, tuberculosis (TB) y lepra, salud medioambiental, malaria y HIV/SIDA fueron las

intervenciones realizadas a continuación. Entre las estrategias de intervención se recurrió al puerta a puerta, tratamiento domiciliario, educación sanitaria y campañas. La participación de la comunidad en la planificación, implementación y supervisión de los proyectos de desarrollo fue generalizada. Entre los recursos disponibles para estas actividades, y que constituyen los recursos potenciales para el proceso de IDC, se encuentran los voluntarios de la comunidad, OBC y ONG. Otros fueron propietarios de inmuebles; asociaciones profesionales, de mujeres y de jóvenes; clubs sociales, organizaciones religiosas y centros de salud disponibles.
Conclusión: Este estudio ha permitido concluir que resulta viable utilizar el proceso IDC para realizar intervenciones sanitarias en comunidades pobres urbanas e indica que en las comunidades existen recursos potenciales de sobra para aplicar esta estrategia.

Translated from English version into Spanish by Sergiman through

TRANSLATORS
WITHOUT BORDERS
