# Supplementary material for: Assessing resources for implementing a community directed intervention (CDI) strategy in delivering multiple health interventions in urban poor communities in Southwestern Nigeria: a qualitative study
Source: Infect Dis Poverty. 2013 Oct 24;2:25. doi: 10.1186/2049-9957-2-25 (PMC4177198; doi:10.1186/2049-9957-2-25)
Supplement: Additional file 2 — FGD and KII guides. [file 2049-9957-2-25-S2.docx]

**Improving Health Care Delivery in Urban Communities in Africa using the Community Directed Intervention Approach.**

**Focus Group Discussion Guide**

**Formative Phase**

**Indicator**: Knowledge of existing health interventions

A**. Information on Existing Interventions**

1. What health interventions exist in your community?

Probe:

- How long have these interventions been in place your community?

- Who is carrying out these interventions?

2. How do you feel about these interventions?

Probe**:**

-Community perception of Priority interventions

- Awareness

- Coverage

- Affordability

- Access

B. **Community Participation and Involvement**

3. How were these interventions introduced in this community?

Probe:

- Community participation/involvement?

- What are the decision making structures in this community?

- Process of participation?

- Which community members are directly carrying out this intervention in your community?

- How were the community members selected to carry out these intervention?

C**. Support for existing interventions**

4.What support did the health system provide for existing interventions

Probe**:**

- Training

- Capacity building

- Supervision

- Incentives to selected community members

5. What support did your community provide for conduct and implementation of the existing interventions?

Probe**:**

-Capacity for training

-Supervision

-Type of contributions provided to support existing interventions

D**. Benefits and Challenges**

6**.** What are the benefits of these interventions to your community?

7. What are your perceptions of the work of the existing community members delivering the interventions

8**.** What factors hinder the work of existing community selected members

9. What challenges do you face as a community due to the implementation of these interventions

E**. General Information**

10. What are the health facilities in this community?

Probe:

-Availability of health care facilities and how they function in the community

-Nearness to health facilities

11**.** How well are these health care facilities satisfying the need of your community?

******************************************************************************

**D. Key Informant Interview Guide for Community Leaders**

**Improving Health Care Delivery in Urban Communities in Africa using the Community Directed Intervention Approach.**

Key Informant Interview Guide for Community Leaders

INSTRUCTION

1. **Who to Interview:** Community leaders. These include
   - 1. Traditional leader
     2. Women Leader
     3. Community development group leader
     4. Major CBO leader
     5. Religious group leaders (both orthodox and traditional)
2. **Sample:** Select the traditional leader, one women leader and one each from any other two groupings in the community. This brings to four the number of community leaders to be interviewed
3. **When:** Administer instrument at formative stage and after intervention

Project Site-------------------------------[__]

District/LGA -----------------------------------[__]

Urban Name __________________[_ ][_ ][__]

Household ID No. [__]

IDENTIFICATION CODE. [__][__][__][__][__]

Enumerator___________________________

Date__________

The Community-Directed Intervention (CDI) approach intends to use the Community as it is used for Ivermectin (Mectizan) delivery in the rural area for the administration of other health interventions in the urban area. In this context, the Community, the Health Services, and other partners have specific roles to perform in order to determine the extent to which the CDI approach can be used to improve health care delivery in poorly served urban population.

Please, would you like to take part and provide information to the following questions? Information provided will be kept strictly confidential.

[If yes, proceed with interview. If No, Stop]

**Position in the Community __________ Name (Optional) --------------------------**

**Number of years as Community Leader __________________**

**Occupation ____________ Sex ­­­­___________________**

**A. Awareness/Perception**

1. What are the health facilities in this community? (Obj. 4)

Probe into:

- 1. Availability of health care facilities and how they function in the community.
  2. Who owns the facilities
  3. Who manages
  4. Who uses
  5. Nearness of health facilities
  6. How well are these health care facilities satisfying the need of this community
  7. Existing health interventions

2. Which types of diseases do you experience in this community?

Probe for

- Most common diseases experienced in this community
- The most serious and severe of these diseases to community members and reasons for perception and prioritization

3. Please describe the decision making structures in this community

Probe for:

- People involved in the decision making
- How the people in the community come to know the decisions
- Problem solving mechanisms within the community
- Have the community been involved in existing health interventions
- Types of existing health interventions involved in
- Level of community involvement ( Probe for involvement at planning, implementation and monitoring)
- Type of support or incentives given to existing health interventions (financial, material, labour)
- Was the incentive or support given a participatory decision?

4. How does the community provide incentives for existing health interventions?

Probe for:

- Type of incentives and frequency
- Effects of incentive

**Key Informant Interview Guide for Partners in Community oriented programmes**

**Improving Health Care Delivery in Urban Communities in Africa using the Community Directed Intervention Approach.**

Key Informant Interview Guide for Partners in Community oriented programmes

**INSTRUCTION:**

**a) Who to Interview:** All focal persons for Partners involved in the existing Interventions in the study site

**Sample:** Select one (1) focal person for each Partnering groups in the study area

**When:** Administer instrument at formative stage and after intervention

1. Which of the health interventions in this community is your Organization/Agency involved in?
2. How did you get involved in the interventions and when?
3. Why did your organization/Agency decide to get involved?
4. What type of support has your Organization/Agency given to the interventions you have mentioned above? (Probe for the nature support)
5. How does your Organization support in related programmes?
6. How does your Organization/Agency view related programmes?
7. How is the attitude your Organization/Agency to related programmes?
8. How is the support provided by your Organization/Agency to related Programmes?
9. Has your organization put in place any **arrangements** for dialogue and problem solving within the partnership?

*********************************************************************

**Key Informant Interview Guide for District /LGA/health Programme Managers/Coordinator**

**Improving Health Care Delivery in Urban Communities in Africa using the Community Directed Intervention Approach.**

Key Informant Interview Guide for District /LGA/health Programme Managers/Coordinator

**INSTRUCTION:**

1. **Who to Interview:** Programme managers for each of the existing intervention in the study Districts/LGAs. Note that study Districts/LGAs include those in both the intervention and control arm in the entire study. This is the same wherever it is encountered in the instruments
2. **Sample:** Select one (1) programme manager per programme in each study District/LGA
3. **When:** Administer instrument at formative and after intervention

**Project Site-------------------------------[__]**

**District/LGA -----------------------------------[__]**

**IDENTIFICATION CODE. [__][__][__][__][__]**

Enumerator___________________________

**Date__________**

Please, would you like to take part and provide information to the following questions. Information provided will be kept strictly confidential.

[If yes, proceed with interview. If No, Stop]

**Name (Optional) ___________________________**

**Sex**  1 Male [ ] 2 [ ]

**Number of years of Experience ____________________________**

**Designation__________**

1. What are the major health issues among the underserved urban populations in this community?
2. Which of these issues are priorities?
3. Why do you perceive these issues to be priority?
4. Why are the other issues which are not priority?
5. What are the activities identified to be carried out on the health care in this community?
6. Which of these activities are being implemented Probe

- Community participation
- Delivery of services – who, how, when
- Supply and procurement
- Linkages
- Threats/challenges

1. What are the strengths and weaknesses of the activities implemented in the community?
2. Why are the other activities not implemented?

Probe for

- Challenges
- Weaknesses

1. In what ways can the delivery of health services among the underserved urban population be improved on in this community

Probe for

- Linkages
- Collaboration
- Support
- Opportunities

1. Could you list the partners and stakeholders involved in the delivery of health interventions in this community?
2. What is the level of involvement of each of the partners and stakeholders in the delivery of health interventions?
3. To what extent are the partners and stakeholders coordinated in delivery of health interventions?
4. How many health personnel do you work with?

Probe for

- Number
- Category
- Distribution

1. What health intervention commodities are available?

Probe for

- Drugs
- IEC materials
- Vaccines
- ITNs
- Others (list)

1. What are the resources available for delivery of health interventions in the community?

**Key Informant Interview Guide for Health Worker Attitude towards the CDI process in poorly served Urban community.**

**Improving Health Care Delivery in Urban Communities in Africa using the Community Directed Intervention Approach.**

Key Informant Interview Guide for Health Worker Attitude towards the CDI process in poorly served Urban community.

**When:** Administer instrument at formative stage and after intervention

1. Which of the health interventions in this community are you involved in?
2. How did you get involved in the interventions, why and when?
3. What type of support have you given to the interventions you have mentioned above? (Probe for the nature support)
4. How is your support related to the programmes?
5. Has your organization put in place any **arrangements** for dialogue and problem solving within the partnership?

*********************************************************************

**IN-DEPTH Interview Guide for Volunteers**

**Improving Health Care Delivery in Urban Communities in Africa using the Community Directed Intervention Approach.**

IN-DEPTH Interview Guide for Volunteers

**BACKGROUND**

Name_______________________________

Position__________________________________

Position prior to current work, length of experience______________________________

What other work do you do for a salary___________________________________

Age______________________________

Gender_______________________________

Place of residence_______________________

Place of work___________________________

**JOB RESPONSIBILITIES**

1. What kind of volunteer work do you do?
2. Where do you do this?
3. Describe a typical day as volunteer?
4. What is the most challenging part of your work? What do you enjoy most in your work?
5. Describe your interactions with the community?
6. Describe your interactions with the health service system? (e.g. supervision)
7. What are the current health programs you are implementing in this urban area?
8. How often do you interact with health services stakeholders?
9. Do you ever have contact with other volunteers or other health related personnel from the community?

PROBE🡪 Can you describe these interactions?

1. How did you get into being a volunteer?
2. How are you motivated for the work you do?
3. What are the challenges you are facing as a volunteer?
4. What suggestions do you have for improving volunteer work

************************************

**Type of instruments and targeted number of interviews or FGDs by instrument**

|  | **Type of interview** | **Level of data collection** | **No of data collection Unit** | **Interviews per data collection Unit** | **Total number of interviews/FGD** |
| --- | --- | --- | --- | --- | --- |
| 1 | Key Informant Interview guide for Programmes Managers | National, regional and district | 3 | 5 | 15 |
| 2 | Key Informant Interview guide for Community /Local Leaders | Community | 10 | 2 | 20 |
| 3 | Key Informant Interview guide for Volunteers | Community | 10 | 2 | 20 |
| 4 | Key Informant Interview guide for Health Care Providers | Health facility Community | 10 | 1 | 10 |
| 5 | Key Informant Interview guide for Partners | National Regional, District. Community | 4 | 2 | 8 |
| 6 | Focus Group discussion Guide for Community | Community | 6 | 2 | 12 |
| 7 | Document Review Check | Nationals Regional District Community | 4 | Minimum of 3 | Minimum of 12 |

## Document Review Guide for National, Sub National, District and FLHF levels

| **Objective(s)** | **Guide questions** | **Document to review** | **Specific sources** | **Level of data collection** |
| --- | --- | --- | --- | --- |
| **1**  Document the delivery process of the existing health intervention in poorly served urban communities  **[Formative]** | 1. List of existing health interventions | - Urban Health office annual health reports - Health facilities records and annual reports - Stakeholders health plans and reports - Community based organizations reports/documents - National demographic survey reports - National Poverty Reduction strategy | Urban health authority office  Stakeholders [NGOs]  Community | National  State/District  Community |
|  | 1. Is there an up-to-date report on the existing interventions? | As above | As above | As above |
|  | 1. From the report, what is the role of the:   (i)community  (ii) /health system/  (iii) stakeholders  in the delivery of the interventions? | As above | As above | As above |
|  | 1. Is there a national policy for the delivery of the interventions? | National health policy | National, State and District health offices | National  State  District |
|  | 1. Is there any written policy guiding implementation of the intervention | National health policy/guidelines | MoH | National  State/District |
|  | 1. Is there a provision for availability, affordability and accessibility of the intervention to the poor? | As above |  | \|As above |
|  | 1. Is there any written guideline for the implementation of the interventions? | National guideline | Health facility  Urban health office  District health office | As above |
|  | 1. Is there any policy on public/private partnership? | - National strategy plan - State, district/ urban health plans | MoH  Urban Health office | As above |
|  | 1. Is there a public/private partnership in the delivery of the intervention? | - State/urban health authority reports - NGOs reports - CBOs reports | MoH National  State/District Health office  Community based organisations |  |
| 3.  To determine the feasibility of the use of CDI in poorly served urban communities in the delivery of health care interventions.  [**Formative**] | 1. What is the current district health budget? | National/State/District/ Urban Health Strategic plan and annual Budget | - MoH/ District/Urban Health – | - National  - District |
|  | 1. How much is allocated to existing community oriented interventions? | As above | As above | - Sub- National  - FLHF |
|  | 1. How much of the allocation is actually used for the existing interventions? | - Annual Reports of National/State/District/urban health department  - Reports of other partners involved in the delivery of interventions. | MoH – National  State and urban health office  NGO head office |  |
|  | 1. How many volunteers on the existing interventions were trained? | - Health facility records - Supervisors’ record | Health facility  Community and health facility |  |
|  | 1. How many of the volunteers dropped out? | Supervisors’ record/checklist | Health facility  Research office |  |
| 4To identify the critical factor that facilitate or hinder the CDI process in poorly served urban communities.  **[Intervention phase]** | [Does the urban health plan incorporate CDI process? | - Urban health authority plan | - MoH – National Strategic Plan  - MoH - Annual reports (Surveillance reports) | - National  - District  - Sub- National |
|  | 1. How many of the urban communities have interventions that incorporate the CDI process? | - Health Plans of other stake holders? | - District Annual reports  - Diseases control work-plans  - Report from health partners supporting | - FLHF |
|  | 1. How many of these communities are involved in this study? | - CDI Study proposal | MoH (bilateral donors, international NGOs eg. UNICEF, WHO, USAID, |  |
|  | 1. Does the drug/product policy/guideline support the distribution of drug by volunteers? | - Drug/product Policy of the MOH/urban health office | - District work-plans  - District / Facility work-plans (annual, quarterly etc)  - Directives / memos etc |  |
|  | 1. How many of the study sites involve in CDI implementation process obtain funds from the health authority for their activities? | - Health Authority - Volunteers - Health Facilities | MoH – National, State, District/ Health facility | District  Health facility level |
|  | 1. How many of the sites utilize the funds provided for implementation of CDI process? | - Health facilities records - Expenditure records of community organizations - Annual reports submitted to Ministry of Health | Health facility  Community based organizations  MoH – District/ urban health office |  |
|  | 1. Do health facilities used for CDI have capacity for adequate storage of drugs and products? | - Architectural plan of Health facilities - Health facility annual stock report | State/District/urban health office |  |
|  | 1. How many of the training scheduled for volunteers were conducted. | - Health facility record - Training reports - Health facility annual repote | Health facility  State/District/urban health office | Health Facility level |
|  | 1. How many volunteers submit their reports on time? | - Volunteers record/report | Volunteers | Community |
|  | 1. How many of the reports/records were complete? | - Volunteers records/reports | Volunteers | Community |
|  | 1. How many volunteers keep accurate records? | - Volunteers records | Volunteers | Community |
| **5.**To determine the effectiveness of the CDI process in the delivery of the health intervention in poorly served urban communities.  **[Intervention phase]** | 1. How many community members are served by the volunteer for each intervention? | - Volunteers’ records | As above |  |
|  | 1. How many activities related to CDI process were performed by the community health volunteers? | - Volunteers’ records/reports - Supervisors’ record | As above |  |
|  | 1. Has there been any shortage of drugs/products on any of the interventions? | Drug/product store inventory at HF and community level | Health facilities  Volunteers |  |
|  | 1. How long was the short shortage? | As above + Supervisors’ record/report  Volunteers’ report | “ |  |
|  | 1. Was the shortage during distribution time? | As above | “ |  |
| 6. To determine the efficiency of the CDI process in the delivery of the health interventions in poorly served urban communities. **[Intervention phase]** | 1. How much was spent on:  - Salaries - allowances - training? | - Urban health authority budget - Research budget - Pay roll - Expenditure reports | District/urban health office | - At district level  - Health facility level |
|  | 1. How many hours were spent by volunteers on CDI activities? | - Volunteers time track sheet | Research office |  |
|  | 1. How much was spend on community meetings, mobilization, supervision | - Supervisors records and reports - Research record - NGOs reports - Activities reports |  |  |
